# Breast cancer in Bosnia and Herzegovina: real-world patterns of diagnosis, treatment, and survival outcomes

**DOI:** 10.1038/s41598-026-54863-9

**Published:** 2026-06-03

**Authors:** Semir Bešlija, Emir Sokolović, Timur Cerić, Anes Pašić, Azra Grebo, Amina Aljić, Layan Mattar, Courtney Freedman, Olena Doroshenko, Olena Mandrik

**Affiliations:** 1 Clinic of Oncology, The University Clinical Center of Sarajevo (CCUoS), Federation of Bosnia and Herzegovina, Bolnička 25, 71000 Sarajevo, Bosnia and Herzegovina; 2 The World Bank Group, external consultant, Washington, USA; 3 The World Bank Group, Washington, USA; 4 Jordanian Royal Medical Services, Amman, Jordan

**Keywords:** Breast cancer, Survival, Patient intervals, System intervals, Diagnostic interval, Treatment outcomes, Treatment compliance, Treatment duration, Bosnia and Herzegovina, Eastern Europe, Cancer, Oncology

## Abstract

Timely diagnosis is critical for improving breast cancer outcomes, especially in resource-limited health systems. This study provides the first real-world evidence from Bosnia and Herzegovina on diagnostic and treatment intervals, adherence, and survival in women with breast cancer. We conducted a retrospective analysis of breast cancer cases diagnosed between 2019 and 2023 at the Clinical Centre University of Sarajevo, the largest cancer center in the country, managing approximately 60% of cases in the Federation of Bosnia and Herzegovina. Most patients (78.1%; 95% CI: 73.8–82.3) were diagnosed through self-referral due to symptoms, with 62.4% of cancers detected at stages 1 and 2. The mean patient and system diagnostic intervals were 38.1 days (95% CI: 23.4–52.8) and 38.6 days (95% CI: 29.9–47.3), respectively, with a total diagnostic interval of 70 days (95% CI: 58.9–82.5). Almost all diagnostic procedures had a waiting time of less than ten days. Among all women undergoing surgery, radical mastectomy was performed in 61% (95% CI: 55–66), while among women with stage 1–2 disease, 55% (95% CI: 48–62) underwent radical mastectomy. Treatment duration averaged 3.7 months for chemotherapy (95% CI: 3.4–4.1) and 0.48 months for radiotherapy (95% CI: 0.26–0.69), with higher compliance for radiotherapy (99.5%; 95% CI: 98–100) than chemotherapy (78%; 95% CI: 72–84). Three-year progression-free survival was 85.7%, and overall three-year survival was 86%. Early breast cancer detection and favorable survival can be achieved even in resource-constrained settings.

**Supplementary Information:**

The online version contains supplementary material available at 10.1038/s41598-026-54863-9.

## Introduction

Breast cancer remains the most frequently diagnosed malignant disease among women in Europe, accounting for approximately one in four of all new cancer cases in the female population.^1^ The pattern of cancer incidence in Bosnia and Herzegovina mirrors that of the European Union (EU) and other high-income countries. This trend is largely driven by an increasingly aging population due to migration patterns as well as increased life expectancy.^2^.

According to the International Agency for Research on Cancer (IARC), Bosnia and Herzegovina recorded 14,265 new cancer cases and 8,590 cancer-related deaths in 2022. In comparison, 10,487 new cases and 7,275 cancer deaths were estimated in 2015, indicating an increasing trend in both cancer incidence and mortality.^3,4^.

Unlike cancers such as lung cancer, where prevention is primarily driven by modifiable risk factors, the reduction of breast cancer mortality depends heavily on early detection and timely effective treatment. The stage of the disease at the time of diagnosis remains one of the strongest predictors of survival, as patients diagnosed at earlier stages have a significantly better prognosis.^5^ Recent analyses indicate that delaying treatment initiation by as little as four weeks can increase the risk of death by more than 10%, emphasizing the critical importance of early detection and timely intervention.^6^.

Despite the well-recognized importance of early diagnosis, countries in Central and Eastern Europe, including those in the Western Balkans such as Bosnia and Herzegovina, face persistent challenges. These include unequal access to timely diagnosis, insufficiently organized screening programs, shortages of oncology specialists, limited access to innovative treatments, and scarce data on breast cancer care and patient outcomes.^7,8^ Although the prevalence of breast cancer is increasing in FBiH, the country still lacks a national cancer control plan, systematic access to cancer registries, and an early detection program due to legislative barriers and financial constraints.^9^.

While mammography screening has been introduced in legislation,^10^ there is no evidence of an organized screening program beyond the cantonal level. Available data suggest that where screening is offered, it is largely opportunistic.^7^ Sarajevo canton is among the few where screening practices have been introduced in the recent years. The University Clinical Center of Sarajevo (CCUoS) serves as the only specialised oncology center in the canton and manages approximately 60% of cancer cases in the FBiH.

This study aims to provide the first real-world evidence from Bosnia and Herzegovina on diagnostic and treatment intervals, adherence, and survival in women with breast cancer, using data collected at the CCUoS. The findings from this analysis will be valuable for other countries in Central and Eastern Europe that share similar healthcare system structures, resource constraints, and challenges in implementing organized screening programs.

## Methods

### Study design

This cross-sectional study was conducted at the CCUoS and involved a retrospective review of medical records of patients with suspected breast cancer redirected to the hospital between January 1, 2019, and December 31, 2022. The Ethical Committee of Clinical Center University of Sarajevo approved the study protocol (Decision No 51-45-1-38465/24). This study was retrospective and non-interventional in design and was based exclusively on previously collected medical records. It did not involve the collection of new biological material or direct patient contact, and all procedures were part of routine clinical practice. All methods were performed in accordance with the relevant guidelines and regulations.

The requirement for written informed consent was waived by the Ethical Committee of the Clinical Center University of Sarajevo due to the retrospective nature of the study and the use of anonymized data.

### Study population

This study included data from women who were hospitalised at CCUoS with a suspected diagnosis of breast cancer during the period 2019–2022.

The sample size for the study population was calculated using the formula for proportions, assuming a margin of error (E) of 0.05, a Z-score of 1.96, and an estimated proportion (p) of 0.4 based on preliminary analysis of the first 60 patient records. The total maximum number of patients with breast cancer admitted to the hospital was assumed to be 5,000.

The sample size formula used was.

$$\:n=\frac{N{Z}^{2}p(1-p)}{{E}^{2}\left(N-1\right)+{Z}^{2}p(1-p)}$$

This calculation resulted in a desirable sample size of 234, which was increased to 368 patients to account for missing data (an average of 35% of inputs were missing in the preliminary analysis of the first 60 hospital records).

### Data collection

Data were collected using a pre-coded form developed in Microsoft Excel to minimize the possibility of incorrect data entry. Data entry was performed by trained junior medical practitioners (AG, AA) and final-year medical students at the Clinic of Oncology, under the supervision of senior investigators (BS, SE, PA). Each entry was subsequently cross-checked by an independent reviewer to ensure accuracy and consistency. In cases where data were incomplete or unclear, the original medical records were re-examined, and missing information was complemented through additional verification or follow-up data collection to ensure dataset completeness. Despite these efforts, complete data were not available for all variables; consequently, the number of observations varies across analyses. Each analysis was performed using all available data for respective variable, with a detailed description to calculation provided in supplementary material 1. For variables on resource use, the assumption was made that no data entrance on any resource -related category assumes that the procedure has not been conducted. For relapse status, we replaced the missing status with a newly diagnosed status unless the relapse has been recorded.

### Statistical analysis

The initial statistical analysis plan was developed prior to data collection and subsequently refined following a review of the first 150 patient records, in parallel with the recalculation of the final sample size.

Data cleaning and validation were performed to ensure consistency and accuracy prior to analysis. Missing data were systematically assessed across all variables, and illogical or inconsistent entries (e.g., diagnosis dates preceding symptom onset or treatment initiation before diagnosis) were checked and corrected where possible. All date variables were standardized to the YYYY-MM-DD format to enable calculation of diagnostic and treatment intervals. Categorical variables were harmonized to account for variations in data entry, and derived variables - such as age at symptom onset and age at diagnosis - were computed based on patients’ reported year of birth and relevant clinical dates.

All analyses were conducted using R software (version 4.4.1). Descriptive statistics were used to summarize the dataset: continuous variables were presented as means and medians with interquartile ranges (IQR), while categorical variables were described as frequencies and proportions. Graphical summaries were produced to illustrate the distribution of key continuous variables, such as age at symptom onset and diagnosis.

Progression-free survival (PFS) and overall survival (OS) were estimated using the Kaplan–Meier method. Survival probabilities were reported at one, three, and five years with corresponding 95% confidence intervals. Further details of the statistical analysis plan, including the R code used for implementation, are provided in the supplementary materials. While the analytical code is available on GitHub [https://github.com/lena-mandrik/Breast_cancer_BiH/], the underlying dataset cannot be shared publicly due to patient confidentiality and ethical restrictions.

## Results

### Description of the population

A total of 368 patient cards were analysed. Among those who reported their residence, 90.7% (*n* = 333; 95% CI: 87.7–93.7) lived in a city, 7.9% (*n* = 29; 95% CI: 5.1–10.6) in a town, and 1.1% (*n* = 4; 95% CI: 0.0–2.2) in a rural area.

Of those who specified their location, 92.4% (*n* = 339; 95% CI: 89.7–95.1) lived in Sarajevo Canton, while 7.6% (*n* = 28; 95% CI: 4.9–10.3) resided in other cantons.

Regarding the pathway to diagnosis, 7.1% (*n* = 22; 95% CI: 4.5–9.8) of women were diagnosed through screening mammography, 8.7% (*n* = 27; 95% CI: 5.8–11.6) through screening ultrasound (US), 78.1% (*n* = 242; 95% CI: 73.9–82.3) via a symptomatic pathway, and 0.3% (*n* = 1; 95% CI: 0.0–0.8) through clinical breast examination. An additional 5.8% (*n* = 18; 95% CI: 3.4–8.2) reported “other” pathways.

Most women first sought care in the public primary healthcare sector, 75.2% (*n* = 182; 95% CI: 69.8–80.6), while 11.2% (*n* = 27; 95% CI: 7.9–14.4) visited private facilities and 12.8% (*n* = 31; 95% CI: 9.4–16.2) went directly to secondary or tertiary care.

The mean age at first symptom presentation was 59.5 years (95% confidence interval [CI]: 57.5–61.5; *n* = 175), and the mean age at diagnosis was 62.2 years (95% CI: 60.4–64.0; *n* = 217).

### Stage at diagnosis

**Fig. 1:**
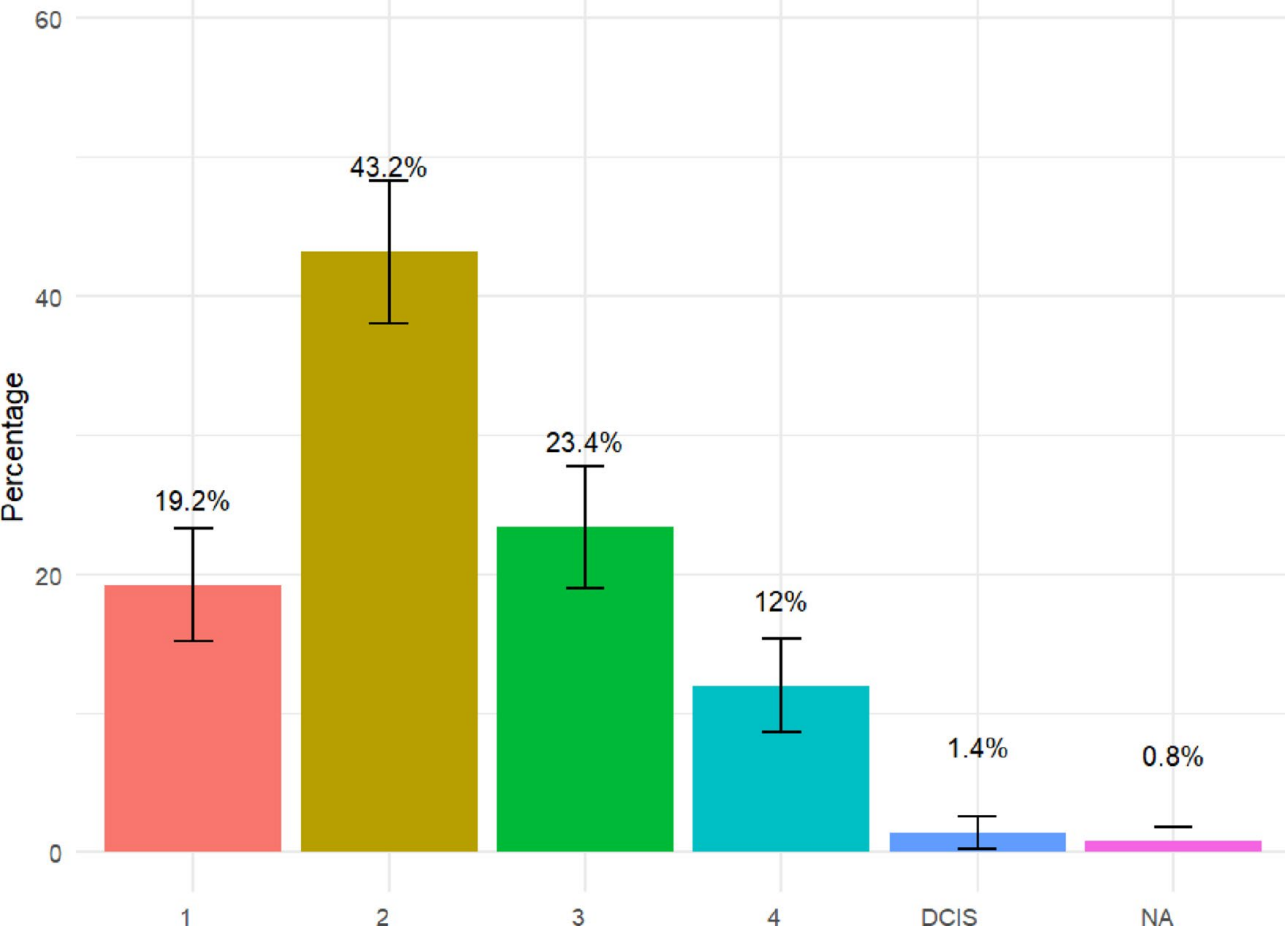
Distribution of stage at diagnosis The Legend: 1–4 Stages by TNM classification system; DCIS – ductal carcinoma in situ; NA -not available.

Most women (43.2%; 95% CI: 38.1–48.3) were diagnosed at stage 2 (Fig. 1). A small proportion (1.4%; 95% CI: 0.2–2.6) were diagnosed with ductal carcinoma in situ (DCIS). The proportion of early (stage 1–2) cancers at the time of diagnosis increased from 0.49 (95% CI: 0.36–0.62) in women diagnosed before 2020 to 0.66 (95% CI: 0.58–0.74) in women diagnosed after 2021.

### Patient and system intervals

On average, patients took 38.1 days (95% CI: 23.4–52.8) to report their symptoms to a general practitioner (GP). Once they did, GPs referred patients to secondary care within 1.3 days (95% CI: 0.59–2.02).

**Fig. 2:**
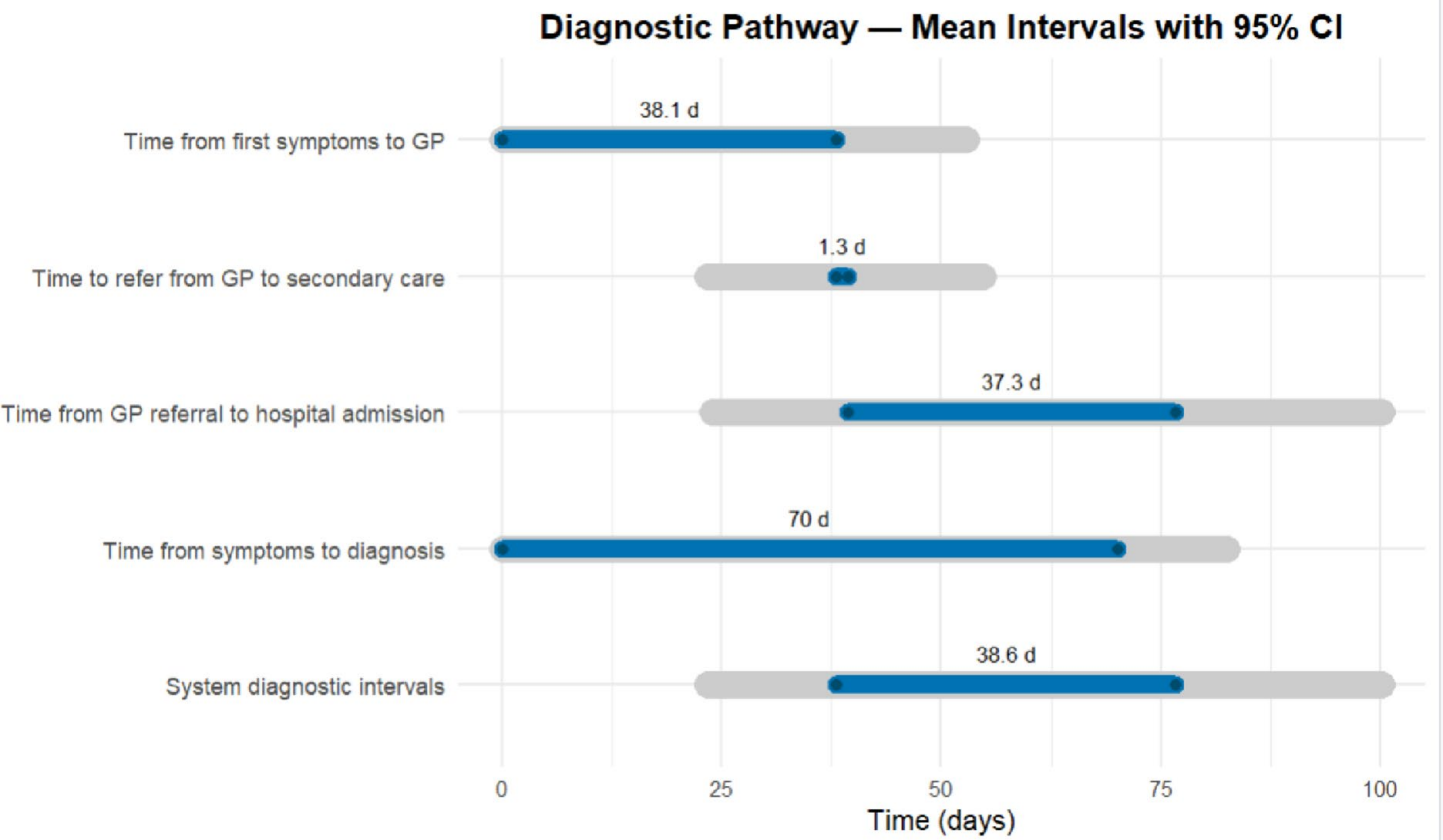
Patient and system diagnostic intervals. The Legend: GP – general practitioner, CI -confidence interval.

**Fig. 3:**
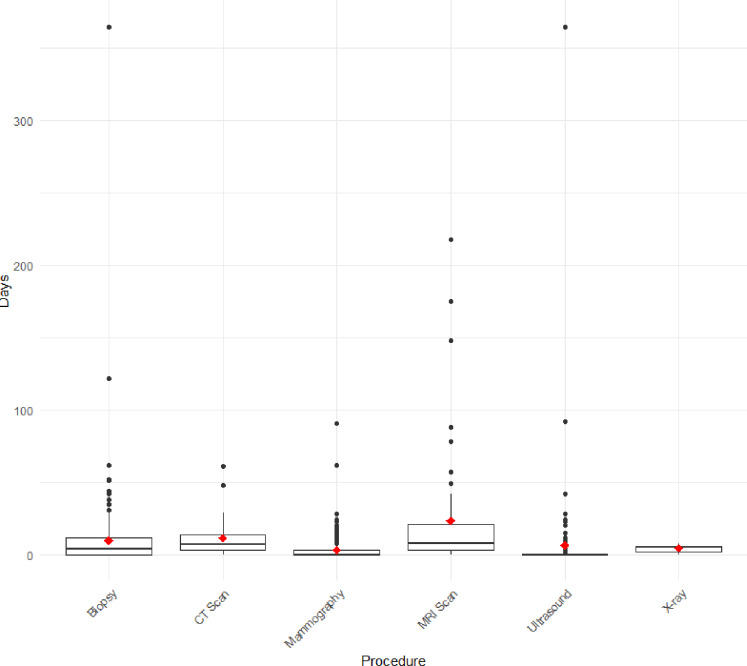
Time to diagnostic procedures.

The interval from GP referral to hospital admission averaged 37.3 days (95% CI: 29.3–45.3). Overall, the diagnostic pathway—from symptom onset to confirmed diagnosis—lasted an average of 70 days (95% CI: 58.9–82.5) (Fig. 2 and 3).

### Diagnostic services received

The waiting time for diagnostic procedures were right-skewed, with most procedures performed within 10 days of being requested. The longest waiting times were observed for biopsy (mean 9.0 days; *n* = 220), CT scan (mean 12.6 days; *n* = 28), and MRI (mean 18.5 days; *n* = 46). Most patients underwent two to three diagnostic procedures. Women who had fewer or more procedures were younger on average (mean age 63.0 years, SD 13.2 for 2–3 procedures; 57.1 years, SD 10.3 for fewer than two; and 55.8 years, SD 8.3 for four or more).

Among all women, 80.0% (95% CI: 75.8–84.0; *n* = 294) underwent a single mammography (MM). Of the total sample, 15.5% (95% CI: 11.8–19.2; *n* = 57) did not receive MM, and 4.6% (95% CI: 2.5–6.8; *n* = 17) underwent a repeat MM. However, if replacement for missing values is not applied, the proportion of women who did not have mammography is much lower (3.6%).

A single ultrasound (US) was performed in 74.5% (95% CI: 70.0–78.9; *n* = 274) of women, while 10.9% (95% CI: 7.5–14.3) had a repeat US. MRI was less common: 19.0% (95% CI: 15.0–23.0; *n* = 70) received one, and only two women had a repeat MRI. All but one woman underwent biopsy; 91.6% (95% CI: 88.7–94.4; *n* = 337) had a single biopsy, while 4.2% (*n* = 15) had repeat biopsies. Core biopsy was the most frequent type (98.9%; *n* = 339), with surgical biopsy used in only 0.9% (*n* = 3). For metastasis detection, 27.5% (95% CI: 22.9–32.0; *n* = 101) underwent one X-ray, and 19.1% (95% CI: 13.9–24.4; *n* = 41) had a CT scan.

### Treatment intervals

**Fig. 4:**
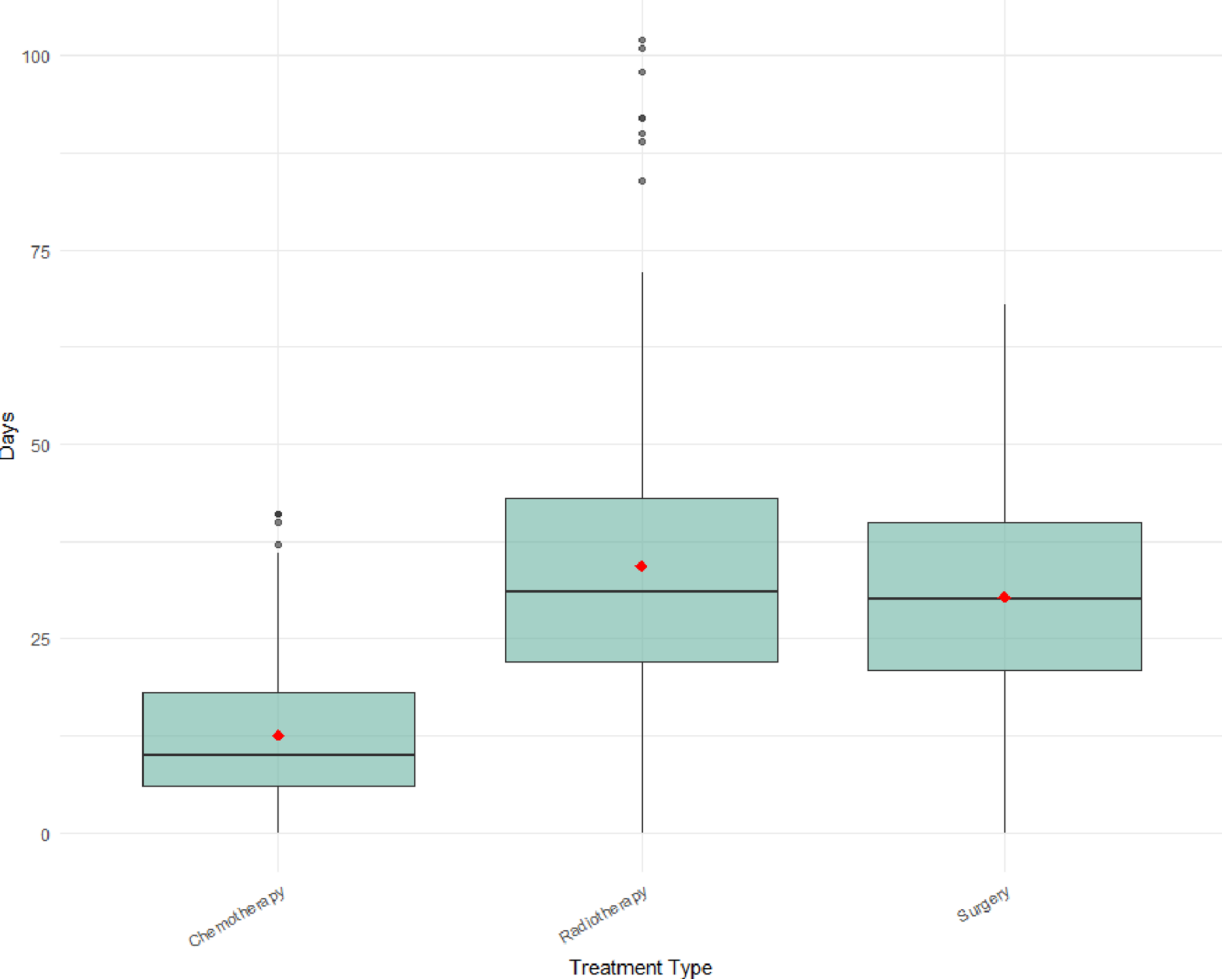
Time to treatment. Legend: Compliance to chemotherapy included neoadjuvant and adjuvant treatment.

The mean time to treatment initiation was 12.5 days for chemotherapy (95% CI 11.2–13.9), 34.3 days for radiotherapy (95% CI 31.4–37.2), and 30.2 days for surgery (95% CI 28.5–32.2) (Fig. 4).

More women underwent radical mastectomy than breast-conserving surgery (60%, 95% CI 55–66 vs. 39%, 95% CI 34–45). Women with early-stage cancer (stage 1–2) were significantly more likely to receive breast-conserving surgery than those with late-stage disease (stage 3–4) (*p* = 0.00073). Nevertheless, 55% (95% CI 48–62) of women with stage 1–2 cancers who underwent surgery still had a radical mastectomy.

### Treatment duration and compliance

Treatment duration was right-skewed, with a mean of 3.7 months (95% CI 3.4–4.1) for chemotherapy and 0.48 months (95% CI 0.26–0.69) for radiotherapy, Table 1. Compliance - defined as completing treatment as prescribed - was higher for radiotherapy (99.5%, 95% CI 98–100) than for chemotherapy that included both neoadjuvant and adjuvant treatments (78%, 95% CI 72–84).

Among 305 women receiving hormonal therapy, 99.3% (95% CI 98.4–100) obtained it free of charge. Of the 79 women who received targeted therapy, the mean number of units administered was 17.6 (95% CI 15.9–19.3; median 18). Only 14.7% (*n* = 10 of 73) paid for one or more units, receiving on average 9.5 units (median 9). The mean duration of hormonal therapy, for those patients who had both the date of initiation and completion recorded, was 40.5 months (95% CI 37.9–43.2; *n* = 242), and for targeted therapy, 13.5 months (95% CI 11.8–15.1; *n* = 73).

**Table 1:** Duration and compliance to therapy.

Parameter	Chemotherapy (*n* = 133)	Radiotherapy (*n* = 184)	Hormonal therapy (*n* = 242)	Target therapy(*n* = 73)
Duration in months, mean and 95% CI	3.7 (3.4; 4.1)	0.48 (0.26; 0.69)	40.5 (37.9;43.2)	13.5 (11.8; 15.1)
Duration in months, median and IQR	4; 3	0;1	50; 39	12; 2
Compliance, proportion and 95% CI (n)	0.78 (0.72 ; 0.84)	0.995 (0.98 ; 1)	NA	NA

### Survival analysis

Survival analysis was performed using the Kaplan–Meier estimator method. Within the first year following diagnosis, the observed overall survival (OS) was 97% (95% CI: 94–99), with three-year survival of 86% (95% CI: 80.5–91.9) and five-year survival of 84% (95% CI: 78.8–91.1).

**Fig. 5:**
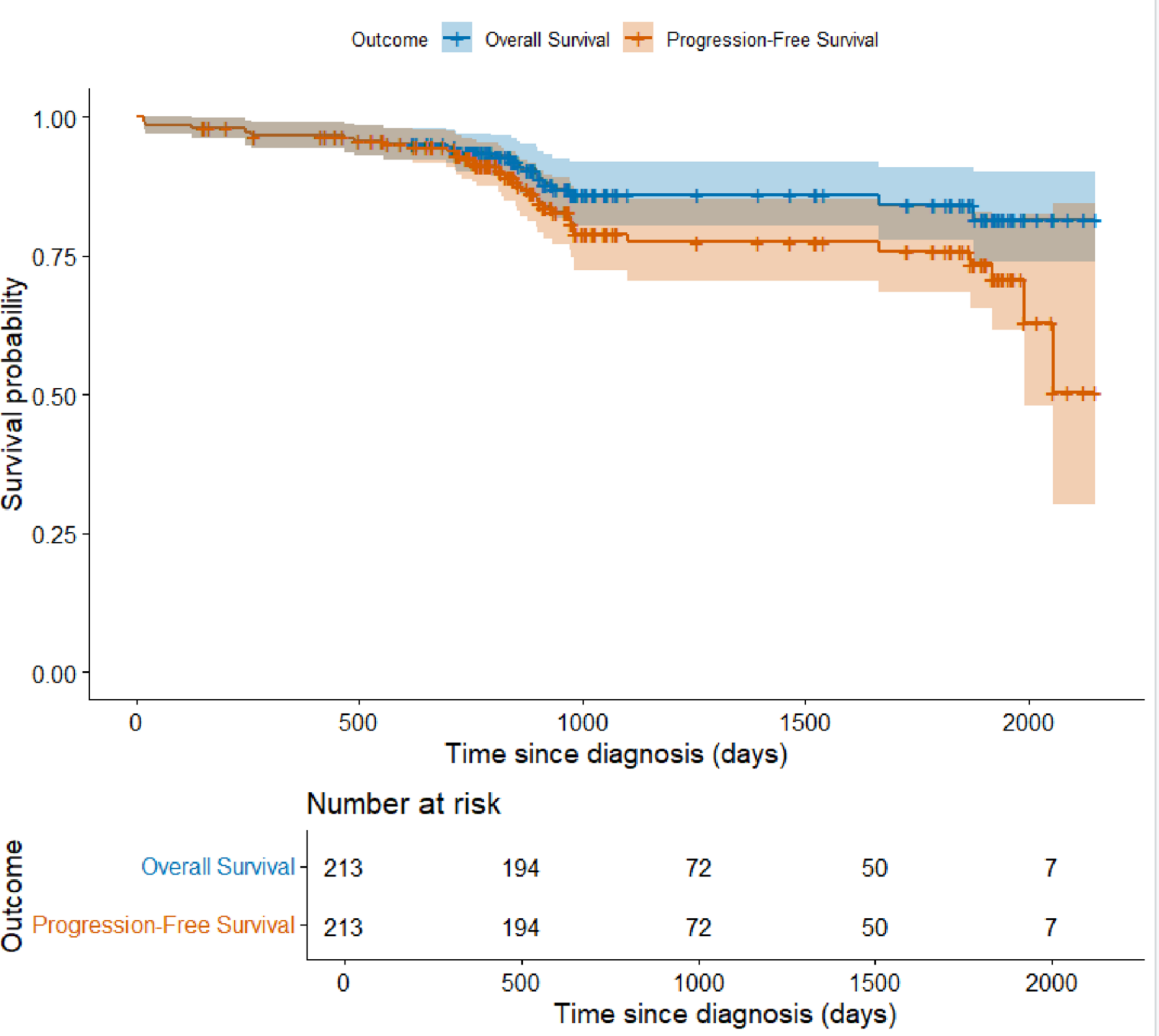
Progression Free and Overall Survival.

The observed progression-free survival (PFS) was 96.7% (95% CI: 94.3–99.1) at one year, decreasing to 78.9% (95% CI: 72.4–86.1) at three years and 75.8% (95% CI: 68.4–84.1) at five years (Fig. 5).

## Discussion

This study aimed to analyse diagnostic and treatment intervals, adherence, and survival in women with breast cancer treated at CCUoS. One of the most important findings was the evidence that there is room for improvement in performance on healthcare system outcomes involving patients’ choices—particularly the patient-related interval to diagnosis, compliance to treatment, and choice of surgery. Variability in diagnostic and treatment decisions, including delays or preferences for specific procedures, further underscores the influence of patient autonomy and perception on treatment timelines. This suggests a need to incorporate patient-centred support into the healthcare system, particularly within secondary and tertiary care, to facilitate informed and timely decision-making.

In our cohort, patients waited 38.1 days from the onset of symptoms to their first consultation with a GP, representing the patient interval in the Aarhus Model of Pathways to Treatment. This interval suggests relatively good symptom awareness and responsiveness, aligning with findings from high-income European countries, where the median ranges around 32 days.^11^.

Recent evidence from England indicates that median diagnostic intervals in breast cancer are approximately 24 days, with additional system-related intervals, including referral and secondary care delays, typically ranging from 11 to 15 days. Delays were found to be longer among patients presenting with less specific symptoms and in settings with limited access to timely diagnostic services. These findings highlight the combined impact of patient- and system-level factors on overall diagnostic delay and emphasize the importance of early symptom recognition and efficient referral pathways.^12^.

Comparable findings were reported in a Serbian multicentre study, where the median patient interval was 4.5 weeks, the system interval 9.2 weeks, and the total diagnostic interval 12.9 weeks, with one in five women waiting longer than 12 weeks before seeking care.^13^ By contrast, countries with well-organized screening and diagnostic systems, such as Slovenia, achieve much shorter intervals—with screening results communicated within 2–7 working days and follow-up diagnostics completed within 4–10 working days.^15^ These comparisons suggest that while some delay in initial help-seeking still exists, the observed patient interval in our population reflects a performance level comparable to European standards.

Several areas for improvement are noted regarding patients’ education. Notably, the proportion of breast-conserving surgery in the FBiH for early-stage (stage 1–2) cancers is relatively low compared to other countries. Surgical options are available and treatment decisions are guided by tumour characteristics; however, the observed pattern cannot be fully explained by the available data. While patient preferences may contribute, this was not directly assessed in the present study.

A range of evidence-based interventions (including “edutainment”, patient navigators, tailored educational materials, and the teach-back method) have been shown to improve breast cancer care outcomes by enhancing patient health literacy and supporting treatment adherence and screening participation.^15–17^ Better integration of primary and secondary healthcare (e.g. active involvement of GPs in managing breast cancer patients’ post-diagnosis) is also essential to improve patient choices and ultimately, clinical outcomes.

Despite limited healthcare capacity, CCUoS achieved an effective diagnostic pathway, with most breast cancer cases in Sarajevo Canton being diagnosed at early stages (1 and 2) during 2019–2023. Similar patterns have been observed in countries where screening has been introduced only recently or is still largely opportunistic, such as Moldova and Armenia.^19,20^ Still, the proportion of early-stage diagnoses is lower than in countries with long-standing national breast cancer screening programs, such as Belarus and Ukraine.^21,21^.

The COVID-19 pandemic temporarily disrupted screening services across Europe, but studies report no lasting increase in late-stage presentations.^23^ A recent global analysis also found a gradual decline in the proportion of metastatic (stage 4) cases and an increase in early-stage diagnoses over the past two decades, particularly in high-resource settings.^24^ Consistent with these observations, in our cohort the proportion of early-stage cancers detected in 2022 and 2023 was higher compared to the pre-2020 period, suggesting that post-pandemic organizational adaptations and heightened patient vigilance may have contributed to earlier diagnosis.

In Sarajevo Canton, the majority of breast cancer patients (78.1%) were diagnosed through self-referral due to symptoms, while only 7% and 9% were diagnosed through screening mammography and ultrasound, respectively—patterns similar to those in Serbia and other countries with limited screening coverage. Nevertheless, the diagnostic system performed efficiently: the mean system interval (symptom presentation to diagnosis) was 38.6 days, compared with a 12-country average of 77.7 days.^12^ The total diagnostic interval in our cohort (70.7 days) was also shorter than the international average (100.8 days), reflecting effective triage, referral, and coordination.

These strengths are mirrored in favourable survival outcomes. The estimated five-year PFS was 77.3% and OS 83.7%. This exceeds rates reported in several Central and Eastern European countries,—e.g. 74.2% five-year OS in Hungary^25^ and 74–78% in Estonia, Romania, Lithuania, and Bulgaria^26^—and suggests that timely diagnosis and coordinated care can yield strong outcomes even in resource-constrained systems.

To sustain progress, a national cancer control plan is essential to guide system development and establish an organised, population-based screening programme. Countries with organised or well-established opportunistic breast cancer screening programmes (i.e. with high screening coverage) demonstrate a larger share of early-stage diagnoses.^10^.

Currently, estimates for breast cancer screening coverage in the Federation of Bosnia and Herzegovina remain extremely low (0.8%)^10^. In our analysis, 7.1% of women were diagnosed through screening, reflecting limited uptake of screening services.

Similar challenges on low screening coverage rate are seen across Eastern Europe - Romania (9%),^26,27^ Hungary (30%),^27,28^ and Latvia (31–47%)^28,29,30^29 with frequent unfavourable combinations of low screening coverage and high inequality reported in Bulgaria, Romania, and a number of Baltic and Mediterranean countries^30,31^—indicating that even established programmes struggle with participation, particularly in deprived and rural areas.

### Strengths and limitations

Several limitations should be acknowledged. First, the retrospective design may have introduced biases related to incomplete documentation and potential inaccuracies in the recorded data.

Second, as the CCUoS is a single tertiary referral centre and a leading institution in Sarajevo Canton, the study population is largely drawn from this region and may not be fully representative of the broader population of Bosnia and Herzegovina. Although the centre receives referrals from other areas, regional differences in access to diagnostic and treatment services may limit the generalisability of the findings. Therefore, the results should be interpreted with caution, particularly when making comparisons with other healthcare systems.

Finally, the survival analysis was not adjusted for competing mortality risks by age, and therefore should not be directly compared with age-standardised or net survival estimates from other countries.

## Conclusion

This study demonstrated that efficiency of breast cancer diagnostic pathways can achieve early breast cancer detection and favorable survival even in resource-constrained settings. The study also highlighted opportunities to strengthen healthcare processes by focusing on patient-related factors emphasising the role of patient education and integrated care.

## Supplementary Material

Below is the link to the electronic supplementary material.

Supplementary Material 1

## Data Availability

The data that support the findings of this study were generated by the authors and are not publicly available due to patient privacy and ethical restrictions. The data are available from the corresponding author upon reasonable request.
