# Supplementary material for: Breast cancer in Bosnia and Herzegovina: real-world patterns of diagnosis, treatment, and survival outcomes
Source: Sci Rep. 2026 Jun 3;16:25379. doi: 10.1038/s41598-026-54863-9 (PMC13473604; doi:10.1038/s41598-026-54863-9)
Supplement: Supplementary file 1 [file 41598_2026_54863_MOESM1_ESM.docx]

**Supplementary 1. Statistical analysis plan**

This Statistical Analysis Plan (SAP) outlines the procedures for cleaning, transforming, and analyzing data collected on patients diagnosed and treated for breast cancer at the University Clinical Center of Sarajevo (CCUoS). The aim of this analysis was to describe real-world diagnostic and treatment intervals, treatment adherence, and follow-up outcomes for women with breast cancer in the Federation of Bosnia and Herzegovina (FBiH). The final analytical sampling used to validate the SAP and calculate the final sample size was 150 patient entries.

***Software***

All data management and statistical analyses were conducted in R (version 4.4.1) using the following packages:

- tidyverse for data wrangling and visualization
- lubridate for date handling
- dplyr and data.table for aggregation and reshaping
- ggplot2 for graphical representation
- survival for exploratory time-to-event analysis (where feasible)

***Data Cleaning and Management***

Data were screened for missingness, logical consistency, and temporal order. Missing values were not imputed for any variable. Illogical entries - such as negative intervals (e.g., treatment start before diagnosis) or implausible dates (e.g., birth year after diagnosis year) - were flagged for exclusion in variable-specific analyses.
All date variables were converted to Date format (YYYY-MM-DD) to enable accurate computation of time intervals. Derived time variables (e.g., time to referral, time from symptom onset to diagnosis) were calculated in days, with results expressed in median and interquartile range (IQR) due to non-normal distributions.

***Standardization of Key Variables***

- **Age at diagnosis:** Calculated as the minimum year among recorded diagnosis year, first admission, or referral year to CCUoS, subtracted from the year of birth. This approach minimized illogical sequences (e.g., treatment preceding diagnosis).
- **Residency:** Categorized both by type (urban vs. rural) and canton. Proportions were calculated relative to the total analytic sample.
- **Stage at diagnosis:** Categorized according to TNM stage (I–IV). Approximately 8% of cases had missing stage data in the initial statistical sample; these were excluded from stage-specific summaries.
- **Referral pathway:** Two recorded inputs were assessed:
  1. **Mode of examination** (symptomatic, screened, or self-examination)
  2. **Level of care** (primary vs. secondary)

For the mode of examination, 37% of data were missing in the initial sampling. Given the implausible distribution (only 10% “symptomatic” vs. 40% “self-examination”), these two categories were merged into a single *“symptomatic cases”* group.

***Approach to the outliers and illogical values***

Outlier detection was conducted using the Interquartile Range (IQR) method, which is a robust approach suitable for continuous data. The first (Q1) and third (Q3) quartiles were calculated, and the IQR was defined as the difference between them (IQR = Q3 − Q1). Values falling below Q1 − 1.5 × IQR or above Q3 + 1.5 × IQR were classified as outliers. This method does not assume a specific distribution and is less sensitive to extreme values, making it appropriate for skewed data. Additionally, illogical values, such as negative time values, were excluded from the calculation of descriptive statistics.

***Derived and Composite Time Intervals***

To address incomplete or inconsistent temporal data, composite indicators were derived:

1. **Time from first medical referral to admission:**
   Calculated as Admission date−Earliest available referral date, where the referral date was defined as either the “symptomatic referral date” or the “referral to CCUoS date.”
2. **Time from symptom onset to first admission:**
   Calculated as Earliest recorded admission or referral date−Symptom onset date.

Entries yielding negative or implausible values (e.g., referral date after treatment start) were excluded from respective analyses. Visual inspection (boxplots and scatter plots) was used to identify outliers, and extreme values were reported but not automatically excluded unless clearly illogical.

***Diagnostic and Treatment Variables***

**Diagnostic Procedures**

Each patient was considered to have received a diagnostic procedure if at least one of the following fields was complete:

- Date requested
- Date conducted
- Test result (positive/negative)

Number of diagnostic procedures was computed both as:

- Mean number per patient across the full cohort, and
- Mean number among patients who had ≥ 1 recorded diagnostic procedure.

Due to outliers (e.g., patient ID 23 with 12 “Other” diagnostic procedures), results were presented as both mean ± SD and median [IQR] to avoid distortion.

Waiting time for diagnostic procedures was calculated only for records with both request and result dates available. Because of limited entries, waiting time distributions were reported descriptively without inferential testing.

Test positivity was defined as the proportion of positive results among all conducted tests. Missing positivity results were assumed to be missing not at random (MNAR), potentially corresponding to negative findings; this limitation was noted in interpretation.

**Treatment Variables**

Each patient was considered to have received a treatment procedure if at least one of the following fields was complete: date of request, date of commencement, type of treatment, length of prescribed treatment, date of completion.

For treatment, median, IQR, and mean waiting times were computed for patients who had both request and completion dates.

Durations were calculated only for patients with valid data, excluding missing or implausible values. To avoid capturing ongoing or incomplete treatments, we included only patients whose last treatment date occurred at least 60 days before their last recorded visit.

For hormonal therapy, discontinuation was defined as the difference between the start date and either discontinuation date or (if missing) the last follow-up date. The duration of hormonal and targeted therapy was estimated for all women who received these treatments, without adjustment for the timing of their last visit. As such, these results reflect current treatment duration rather than compliance

***Follow-Up Outcomes and Survival***

The time between the last two recorded follow-up visits was computed for each patient. In cases where the recorded order of dates resulted in negative values, the sequence was reversed to correct likely entry errors. Differences in mean and median follow-up intervals between relapsed and non-relapsed patients were explored descriptively. Those patients who had missing status, were assumed to be newly diagnosed patients.

Overall survival (OS) and progression-free survival (PFS) were estimated using the Kaplan–Meier (KM) method, implemented in R (version X.X.X) with the survival and survminer packages. For each patient, the date of diagnosis, date of death, and date of last visit were converted to standard date formats (YYYY-MM-DD). Survival time was defined as the number of days from the date of diagnosis to either death (event = 1) or last recorded follow-up (event = 0, censored). The cohort was restricted to patients diagnosed between 2019 and 2022 inclusive to ensure consistent follow-up periods. KM survival functions were fitted using the survfit() function with no covariates (Surv(Time_days, Status_dead) ~ 1), providing non-parametric estimates of the survivor function. Survival probabilities were summarized at 1, 3, and 5 years (365, 1095, and 1825 days). Confidence intervals for the survival function were calculated using Greenwood’s formula, and curves were visualized with 95% confidence bands and risk tables using ggsurvplot().

For progression-free survival (PFS), the event was defined as either disease progression, relapse, metastatic recurrence, or death from any cause, coded as Event_PFS = 1. Patients without such events were censored at the date of their last recorded follow-up. The time-to-event variable (Time_PFS_days) was calculated as the number of days between diagnosis and either event or censoring. Similar to OS, KM estimates of the PFS function were generated using survfit(), with summary probabilities at 1, 3, and 5 years. PFS curves were plotted with 95% confidence intervals and accompanying risk tables to display the number of patients at risk at each time point.

The KM estimator was chosen for its suitability in small samples and its non-parametric treatment of censored data, allowing survival probabilities to be estimated without distributional assumptions. Although formal covariate adjustment (e.g., Cox proportional hazards modeling) was not performed due to limited sample size and missing data, the KM curves provide an empirical description of survival and progression patterns in this cohort.

***Data and Code Availability***

All data cleaning and analytical scripts were implemented in R and are available in the open access GiT platform [add access here]. Access to the underlying dataset may be potentially provided in deidentified form, subject to institutional and ethical approvals from the University Clinical Center of Sarajevo.

**Supplementary Table S2. Complete cases used in calculation of different variables**

| *Denominators used in calculations of the outcomes* | N | % of the total sample size |
| --- | --- | --- |
| Total sample size | 368 | 100% |
| Residence (rural/urban) | 367 | 100% |
| Residence (canton) | 369 | 100% |
| Pathway to diagnosis | 310 | 84% |
| Pathway to diagnosis (public versus private) | 242 | 66% |
| Age at symptoms | 175 | 48% |
| Age at diagnosis | 217 | 59% |
| Stage at diagnosis | 356 | 97% |
| *Patient and system intervals* |  |  |
| Symptoms to First Referral | 98 | 27% |
| Referral to GP Referral | 124 | 34% |
| GP Referral to Admission | 156 | 42% |
| Symptoms to Diagnosis | 120 | 33% |
| *Diagnostic services received* |  |  |
| Waiting time for: |  |  |
| Biopsy | 220 | 60% |
| CT | 28 | 8% |
| MRI | 46 | 13% |
| *Other outcomes* |  |  |
| Reported resource use (MM, MRI, biopsy, X-ray, CT) | 368 | 100% |
| *Treatment duration* |  |  |
| Chemotherapy | 133 | 36% |
| Radiotherapy | 184 | 50% |
| Hormonal therapy | 242 | 66% |
| Target therapy | 73 | 20% |
| *Other treatment outcomes* |  |  |
| Proportion of women receiving hormonal therapy for free | 305 | 83% |
| Number of units of target therapy administered | 79 | 21% |
| *Survival analysis (year of diagnosis > = 2019 and < = 2022)* | 213 | 58% |
